# Predominance of Ferroptotic Cell Death Mechanisms in Substantia Nigra Neurodegeneration in Parkinson's Disease

**DOI:** 10.1002/ana.78202

**Published:** 2026-03-10

**Authors:** Yue Jing Heng, Anusha Jayaraman, Richard Reynolds, Jia Nee Foo

**Affiliations:** ^1^ Lee Kong Chian School of Medicine Nanyang Technological University Singapore Singapore; ^2^ National Neuroscience Institute Singapore Singapore; ^3^ Department of Brain Sciences, Faculty of Medicine Imperial College London London United Kingdom

## Abstract

**Objective:**

The extent of neuronal loss in Parkinson's disease (PD) and the pathogenic processes underlying neuronal dysfunction and loss remain poorly understood. Here, we analyzed the expression of key molecules representing different cell death signaling pathways and their association with Lewy pathology, dopaminergic (DA) neuron loss and stage of PD progression in human postmortem brain tissue.

**Methods:**

We performed neuropathological and molecular analyses on 47 postmortem substantia nigra (SN) tissue samples from PD cases and healthy controls to investigate neuronal cell death pathways.

**Results:**

An average loss of 54% of dopaminergic neurons was found in the SN of PD cases, which correlated strongly with PD Braak staging. The apoptosis markers, cleaved subunits of caspases 3 and 8, were absent. Levels of the active necroptosis kinase, phosphorylated RIPK3 (pRIPK3), were significantly increased in advanced‐stage PD. Although phosphorylated MLKL (pMLKL) levels were not significantly different, both active markers were detected in small numbers of PD neurons by immunofluorescence, suggesting focal necroptotic pathway activation. In contrast, evidence for ferroptosis was more pronounced, particularly in advanced‐stage PD. This was supported by significantly increased transferrin receptor 1 (TFR1) protein levels and significantly decreased glutathione peroxidase 4 (GPX4) RNA and protein levels.

**Interpretation:**

Our findings implicate ferroptosis, and to a lesser extent necroptosis, in PD neuronal death, with ferroptosis potentially playing a larger role in advanced disease. We propose a “2‐hit” model where early synucleinopathy‐driven insults are amplified in advanced disease by a neuromelanin‐iron‐driven feedback loop, establishing ferroptosis as the predominant cell death mechanism. This stage‐dependent shift provides critical insights into PD pathogenesis and suggests distinct therapeutic windows for neuroprotection. ANN NEUROL 2026;99:1415–1427

Parkinson's disease (PD) is pathologically defined by the progressive loss of dopaminergic (DA) neurons in the substantia nigra pars compacta (SNpc) and the presence of intracytoplasmic Lewy bodies, primarily composed of aggregated α‐synuclein.^1^ The accumulation of this protein is associated with a cascade of pathogenic events, including oxidative stress, mitochondrial dysfunction, and neuroinflammation, which ultimately culminate in neuronal death.^2^, ^3^ The selective vulnerability of SNpc neurons^4^, ^5^, ^6^ underlies the characteristic motor symptoms of PD, but significant neuronal loss—estimated at 30 to 70%—has already occurred by the time of clinical diagnosis.^5^, ^7^, ^8^, ^9^ While neuronal loss and clinical progression are closely linked,^10^ the specific molecular mechanisms underlying DA neuron death in the human PD brain remain poorly defined.

Apoptosis has long been considered a primary mechanism of neuronal loss based on studies in toxin‐induced animal models of PD, where apoptotic features and caspase activation are prominent.^11^ However, evidence from human postmortem PD studies is conflicting; while some reported increased levels of apoptosis‐related proteins,^12^, ^13^ many failed to detect classical morphological or molecular hallmarks of apoptosis, such as nuclear condensation or activated caspases, in SNpc DA neurons.^13^, ^14^, ^15^, ^16^, ^17^, ^18^ These discrepancies suggest that additional non‐apoptotic mechanisms are involved in PD pathogenesis.

This has led to increased interest in alternative regulated cell death pathways in neurodegenerative disorders. Necroptosis, a pro‐inflammatory pathway dependent on receptor‐interacting serine/threonine kinases 1 and 3 (RIPK1/3) and their substrate mixed lineage kinase domain‐like protein (MLKL),^19^ has been implicated in Alzheimer's disease (AD),^15^, ^20^ multiple sclerosis (MS),^21^ and amyotrophic lateral sclerosis (ALS).^22^ Given the prominent neuroinflammatory component in PD, necroptosis represents a plausible candidate mechanism for neuronal loss.

The role of ferroptosis, an iron‐dependent form of cell death driven by lipid peroxidation,^23^, ^24^ has also gained significant attention in the context of PD.^25^ The pathological environment of the PD SNpc exhibits features of ferroptosis susceptibility^26^: elevated ferrous iron (Fe^2+^) content,^27^, ^28^ increased lipid peroxidation products,^29^ and decreased levels of glutathione peroxidase 4 (GPX4),^30^ the key enzyme that neutralizes lipid peroxides. Furthermore, genetic links, such as the role of the familial PD gene *DJ‐1* in regulating ferroptosis, strengthen this connection.^31^

To date, few studies have comprehensively investigated these distinct cell death pathways in parallel within a well‐characterized cohort of human postmortem PD brains. Consequently, critical knowledge gaps remain regarding the relative contribution of each pathway, their correlation with the progression of Lewy pathology, and the specific stage of the disease at which they become active. To address these gaps, we conducted a detailed neuropathological and molecular analysis of apoptosis, necroptosis, and ferroptosis markers in postmortem SNpc samples from PD cases across intermediate and advanced Braak stages, as well as from pathologically healthy controls.

## Materials and Methods

### Human Tissue Samples

Snap‐frozen postmortem substantia nigra tissues were sourced from the Multiple Sclerosis and Parkinson's Tissue Bank (Imperial College London) and the South West Dementia Brain Bank (University of Bristol). Samples were obtained from 23 PD cases and 24 pathologically healthy control cases. Among the tissue from 24 control subjects, 12 were provided as tissue blocks suitable for all our analyses, whereas another 12 controls were supplied as tissue shavings for RNA and protein extraction, which could not be used for immunostaining procedures. All neuropathological assessments, including Braak staging based on α‐synuclein immunohistochemistry, were performed and provided by the source brain banks in accordance with the standardized international criteria used by brain banks.^32^, ^33^ Postmortem donation occurred with fully informed consent under ethical approval granted by the National Research Ethics Committee (08/MRE09/31 and NHS REC No 18/SW/0029). The project was also approved by the Nanyang Technological University Institutional Review Board (IRB; IRB‐2018‐09‐052 and IRB‐2016‐12‐029). Detailed demographic and neuropathological information are presented in Supplementary Table S1.

### RNA Extraction and Reverse Transcription Quantitative Polymerase Chain Reaction

Total RNA was extracted from SNpc tissue using the PureLink RNA Mini kit (Life Technologies). The reverse transcription quantitative polymerase chain reaction (RT‐qPCR) was performed using the iTaq Universal SYBR Green One‐Step kit (Bio‐Rad) on a StepOnePlus System (Applied Biosystems). Commercial PrimePCR primers (Bio‐Rad; Supplementary Table S2) were used. Relative mRNA expression was determined using the 2^−ΔΔCT^ methodology,^34^ with normalization to *XPNPEP1* ^35^ and subsequently to total neuronal density (HuC/D+ neurons/mm^2^) for each sample to account for the neuronal loss observed in PD cases compared with the controls.

### Protein Extraction and Western Blotting

SNpc tissue was homogenized in RIPA buffer (Thermo Scientific) with inhibitors. Protein concentration was determined using the BCA assay (Thermo Scientific). Then, 35 μg of lysate per sample was separated on 4 to 12% Bis‐Tris gels (Thermo Scientific) and transferred to PVDF membranes. Blots were incubated with primary antibodies (Supplementary Table S3) overnight, followed by HRP‐conjugated secondary antibodies (Supplementary Table S4). Proteins were detected via ECL (Thermo Scientific) on a Chemidoc Imager (Bio‐Rad). Reprobing was performed to allow the same blot to be probed for different target proteins. The membranes were washed in TBS‐0.1% Tween 20 (TBS‐T) buffer (Bio‐Rad) and incubated protein‐side up in the stripping buffer (Takara) with agitation, for 20 minutes at room temperature. The membranes were subsequently washed in TBS‐T buffer (Bio‐Rad) and blocked with EveryBlot blocking buffer (Bio‐Rad) before immunodetection was performed using a new antibody (see Supplementary Table S3). Band intensities were quantified using ImageJ software (https://ij.imjoy.io/; Rasband, 1997–2015), according to the protocol written by Davarinejad^36^ normalized to GAPDH, and subsequently to total neuronal density (HuC/D+ neurons/mm^2^) to account for the neuronal loss observed in PD cases compared with controls.

### Immunohistochemistry and Immunofluorescence

Immunostaining was performed on 10‐μm cryosections. For DAB staining, sections were fixed in 10% neutral buffered formalin, incubated with primary antibodies (see Supplementary Table S3), and developed using the ImmPRESS HRP polymer reagent and ImmPACT‐DAB substrate kits (Vector Laboratories). For immunofluorescence, the sections were incubated with primary antibodies followed by Alexa Fluor‐conjugated secondary antibodies (see Supplementary Table S4) and mounted with Vectashield Vibrance with DAPI (Vector Laboratories). The pan‐neuronal marker NeuN was used to identify apoptosis and necroptosis in any neuron, whereas the dopaminergic marker tyrosine hydroxylase (TH) was used to specifically investigate ferroptosis within this vulnerable population. Images were acquired on a Zeiss Axio Observer 7 microscope (widefield inverted epifluorescence, Plan‐Neofluar 40×/1.3 Oil DIC objective) and analyzed using ZEN 2.3 software (Zeiss). Slides were stored at 4°C in the dark.

### Image Acquisition and Processing

Whole slide scanning with the Zeiss Axio Scan.Z1 (ZEISS, Germany) slide scanner was used to digitize immunohistochemistry slides stained for TH and neural Hu (HuC/D). It was optimized for brightfield samples and sections were imaged using the 20×/0.8 M27 Plan‐Apochromat objective. The image files were processed using the ZEN Blue 2.3 software. Positive cell densities were quantified using QuPath version 0.2.0 software's “Positive Cell Detection” tool. We limited the analysis to immunostained features with an area > 60 μm^2^ and > 250 μm^2^ to identify HuC/D‐positive (HuC/D+) and TH‐positive (TH+) neurons, respectively. The regions of interest were selected along strict anatomical boundaries. For TH+ neuron count, we delineated the border of the SNpc which was defined by the presence of large, closely packed, TH‐immunoreactive cell bodies and axons. The demarcated region of interest was then superimposed on the HuC/D‐immunostained sections of the same individual, where the counting level and section orientation were identical, to facilitate the quantitation of HuC/D+ neurons. The total number of cells is given as neuronal density/mm^2^.

### Statistical Methods

Non‐parametric tests were used for all analyses under the acknowledgement that the sample sizes might be insufficient to reliably detect significant deviations from normality via formal testing. The Mann–Whitney test was used for 2‐group comparisons, and the Kruskal–Wallis test with Dunn's post hoc test was used for 3 or more groups. PD cases were stratified into intermediate‐stage (Braak III–IV) and advanced‐stage (Braak V–VI). Correlations were assessed using Spearman's rank correlation. Statistical significance was set at a 2‐tailed *p* < 0.05.

## Results

### Tissue Characteristics

Samples were obtained from 23 PD cases (13 men and 10 women; median age of death = 80 years, range = 63–89 years; Braak stages III–VI) and 24 pathologically healthy control cases (11 men and 13 women; median age of death = 86 years, range = 69–94 years), with a mean (±SD) postmortem delay of 26 (±19) hours. The clinical characteristics of these samples are provided in Supplementary Table S1. All pathologically healthy controls did not display alpha‐synuclein immunoreactivity in any of the stained brain sections. The remaining samples were comprised of PD cases that showed varying degrees of alpha‐synuclein pathology and were categorized according to the established PD Braak staging scheme,^32^, ^37^, ^38^ based on internationally validated criteria provided in pathological reports by consultant neuropathologists within the source brain banks. Thirty‐five percent of the PD cases (n = 8) displayed moderate alpha synuclein pathology (PD Braak stages III–IV), whereas the remaining 65% (n = 15) displayed severe alpha synuclein pathology (PD Braak stages V–VI).

### Significant Neuronal Loss in PD Substantia Nigra

**FIGURE 1:**
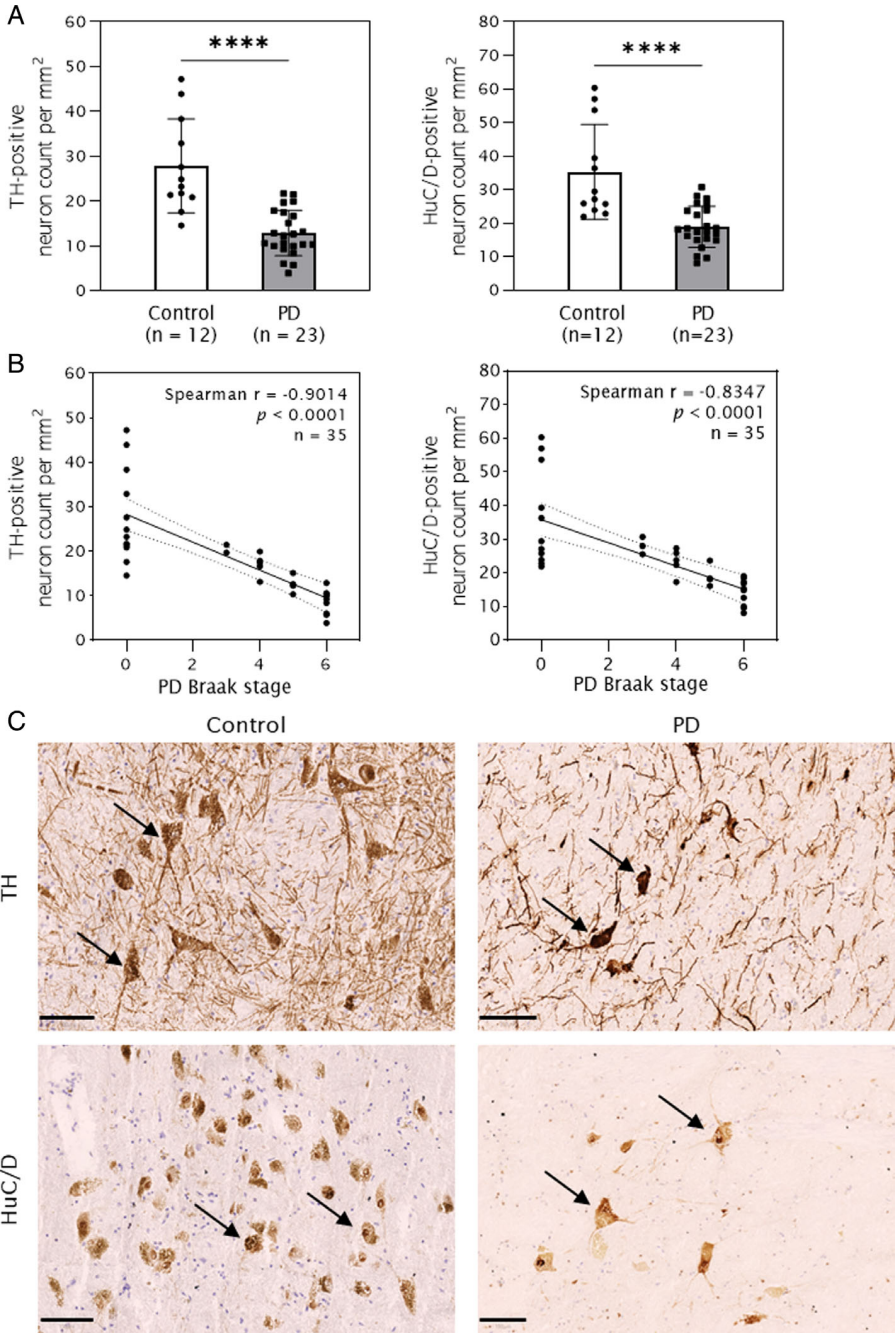
Neuronal density of substantia nigra pars compacta (SNpc) in Parkinson's disease (PD) cases (n = 23) and age‐matched, pathologically normal controls (n = 12). Data are presented as neuron count per mm^2^ (mean ± SD). Each data point represents a sample. (A) A significant reduction of tyrosine hydroxylase (TH)‐and HuC/D‐immunoreactive neurons is observed in PD cases compared to controls (****p < 0.0001). (B) Stratification of samples by PD Braak‐stage shows that the higher the Braak stage, the greater the extent of TH‐positive (*r* = −0.9014) and HuC/D‐positive (*r* = −0.8347) neuronal loss observed. Upper right corner indicates *r* (Spearman correlation coefficient), *p* (associated *p* value) and n (sample size). (C) Representative photomicrographs of diaminobenzidine (DAB) immunohistochemical staining show dopamine neurons labelled by TH (*top row, black arrows*) and total neurons labeled by HuC/D (*bottom row, black arrows*). Hematoxylin was used as a nuclear counterstain in all snap‐frozen tissue sections. All images were captured at 40× magnification. Scale bar = 100 microns.

**FIGURE 2:**
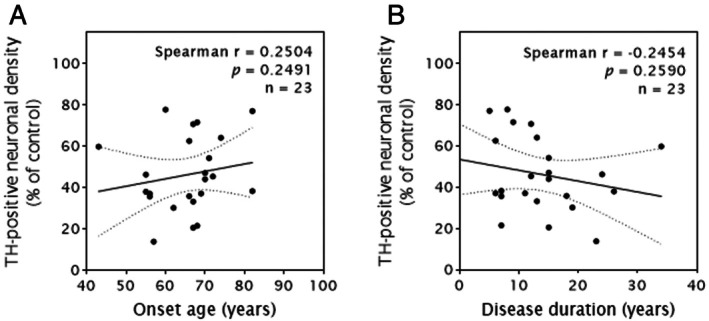
Scatterplot and the corresponding simple linear regression line with 95% confidence intervals showing the relationship between clinical data—(A) onset age, (B) disease duration—and dopamine neuron loss in the substantia nigra pars compacta (SNpc) of Parkinson's disease (PD) cases (n = 23). Each point represents one PD case. Upper right corner indicates *r* (Spearman correlation coefficient), *p* (associated *p* value) and n (sample size).

We observed a significant loss of both total (HuC/D+) and dopaminergic (TH+) neurons in the SNpc of PD cases (n = 23) compared with age‐matched controls (n = 12; *p* < 0.0001; Fig 1A). On average, PD cases exhibited a 46% loss of total neurons and a 54% loss of DA neurons. This neuronal loss was highly inversely correlated with PD Braak stage for both TH+ (*r* = −0.9014) and HuC/D+ (*r* = −0.8347) neurons (Fig 1B). No significant association was observed between neuronal loss and age at onset or disease duration (see Fig 2). Nevertheless, a linear regression analysis of relative DA neuronal density per mm^2^ in the SNpc with extrapolation to time of PD onset revealed an intercept at approximately 60% of control values, which corresponds to an estimated 40% mean DA neuron loss at clinical diagnosis (see Fig 2B).

### No Evidence of Active Apoptotic Signaling in PD Brain

**FIGURE 3:**
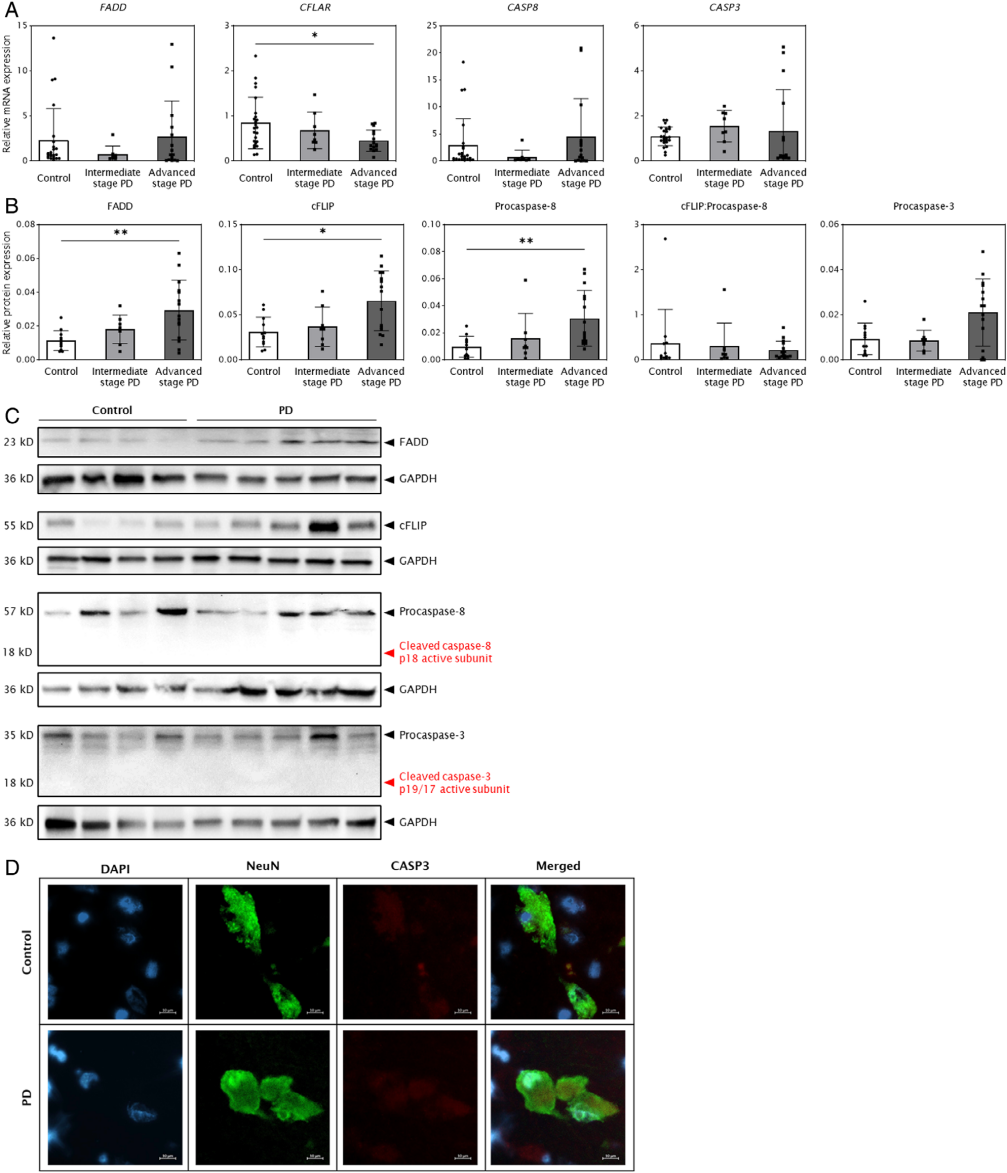
Apoptosis is not the primary cell death mechanism in Parkinson's disease (PD) substantia nigra pars compacta (SNpc). PD cases are grouped as intermediate‐stage (Braak stages III and IV; n = 8) and advanced‐stage (Braak stages V and VI; n = 15). Data are presented as mean ± SD with Bonferroni adjusted *p* value based on Dunn's Test for multiple comparisons (*p < 0.05; **p < 0.01). (A) Analysis of relative mRNA levels of the *FADD*, *CFLAR*, *CASP8*, and *CASP3* genes in the SNpc in controls (n = 24) and PD cases (n = 23), normalized to *XPNPEP1* reference gene. (B) Quantification of FADD, c‐FLIP, procaspase‐8 and procaspase‐3 protein levels in PD (n = 23) and controls (n = 12), normalized to GAPDH and total neuronal density (neurons/mm^2^). (C) Representative Western blots of FADD, c‐FLIP, procaspase‐8, and procaspase‐3. No cleaved caspases were detected (*highlighted in red*). (D) Representative double‐labeling immunofluorescence microscopy of the neuronal nuclear protein NeuN (*green fluorescence*) and activated caspase 3 (*red fluorescence*), with DAPI‐counterstained nuclei (*blue fluorescence*) in snap‐frozen postmortem human SNpc brain sections using an antibody which detects exclusively cleaved caspase 3. No CASP3‐positive neurons were detected. Scale bar = 10 microns.

Analysis of mRNA expression of the key molecular players of caspase‐dependent apoptotic signaling, namely Fas‐associated protein with death domain (*FADD* mean ± SD Control = 2.28 ± 3.52, intermediate [Int] = 0.77 ± 0.88, advanced [Adv] = 2.67 ± 3.98, *p* = 0.4418), caspase‐8 (*CASP8* Control = 2.90 ± 4.91, Int = 0.74 ± 1.27, Adv = 4.42 ± 7.11, *p* = 0.2339) and caspase‐3 (*CASP3* Control = 1.09 ± 0.41, Int = 1.55 ± 0.70, Adv = 1.34 ± 1.83, *p* = 0.0678) did not show significant changes in PD brains compared with controls (Fig 3A). However, mRNA expression level of *CFLAR* (CASP8 and FADD‐like apoptosis regulator), which encodes the endogenous caspase‐8 inhibitor c‐FLIP (cellular FLICE‐like inhibitory protein), was significantly decreased by 1.9‐fold in advanced‐stage PD (Control = 0.84 ± 0.57, Adv = 0.45 ± 0.24, Dunn's adjusted *p* = 0.0432; Fig 3A).

The normalized protein levels of FADD (mean ± SD Control = 0.01 ± 0.01, Adv = 0.03 ± 0.02, *p* = 0.0067), c‐FLIP (Control = 0.03 ± 0.02, Adv = 0.07 ± 0.03, *p* = 0.0186) and procaspase‐8 (Control = 0.01 ± 0.01, Adv = 0.03 ± 0.02, *p* = 0.0072) were significantly increased in advanced‐stage PD compared with controls by 2.6‐fold, 2.1‐fold, and 3.1‐fold, respectively (all *p* values Dunn's adjusted; Fig 3B). However, no significant changes were observed in the cFLIP: procaspase‐8 ratio (Control = 0.37 ± 0.75, Int = 0.30 ± 0.51, Adv = 0.22 ± 0.20, *p* = 0.6617) and the protein level of procaspase‐3 (Control = 0.01 ± 0.01, Int = 0.01 ± 0.005, Adv = 0.02 ± 0.01, *p* = 0.0820; see Fig 3B). Furthermore, no expression of activated cleaved caspase‐3 was observed, either by Western blot (Fig 3C) or immunofluorescence staining (Fig 3D), and no expression of the active cleaved p18 subunit of caspase 8 that activates the downstream pathway of apoptosis was present (see Fig 3C).

### Limited and Focal Evidence of Necroptotic Signaling

**FIGURE 4:**
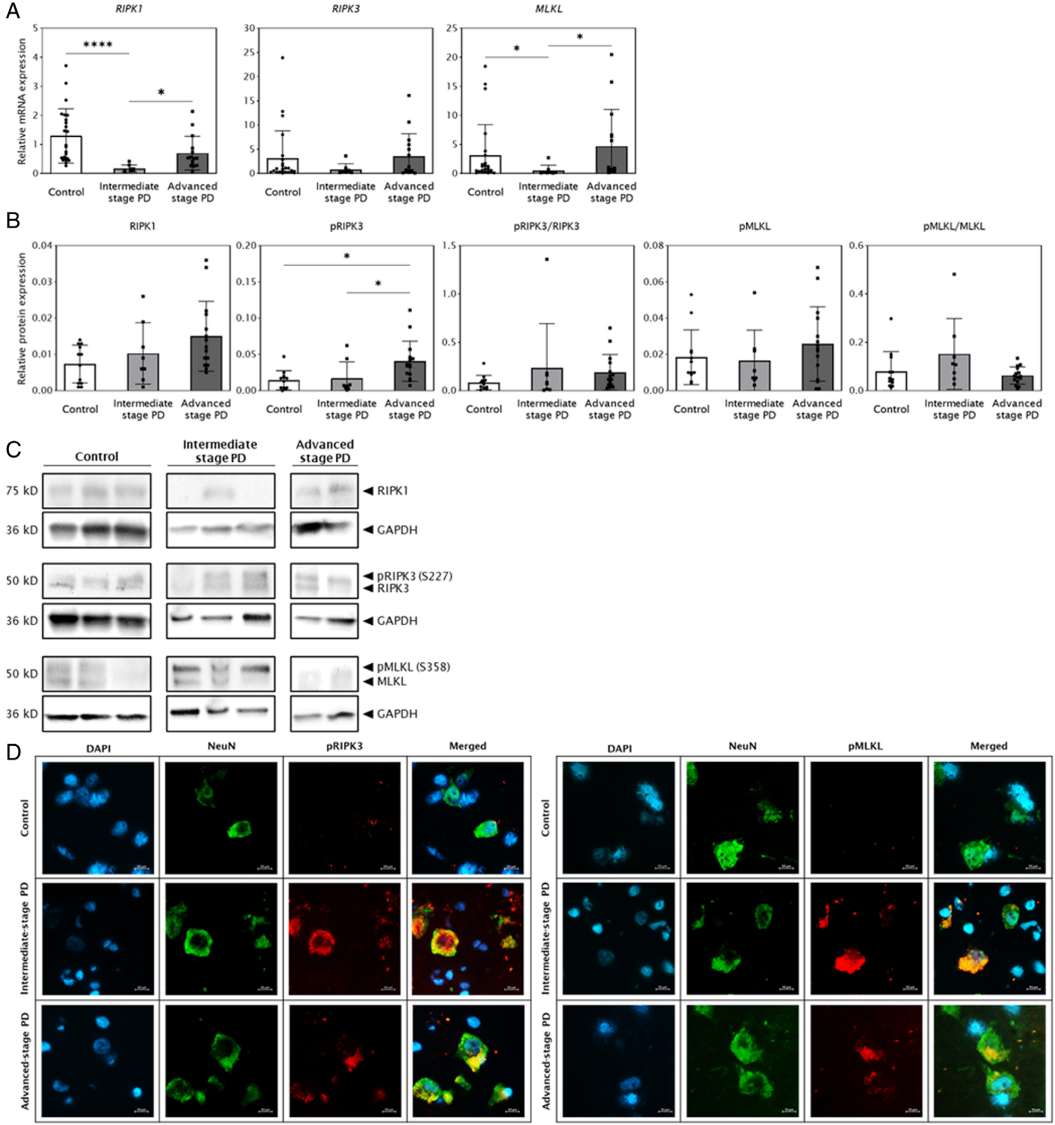
Necroptotic signaling is downregulated in Parkinson's disease (PD) substantia nigra pars compacta (SNpc). PD cases are grouped as intermediate‐stage (Braak stages III and IV; n = 8) and advanced‐stage (Braak stages V and VI; n = 15). Data are presented as mean ± SD with Bonferroni adjusted *p* value based on Dunn's Test for multiple comparisons (*p < 0.05, ****p < 0.0001). (A) Analysis of relative mRNA levels of the *RIPK1*, *RIPK3*, and *MLKL* genes in the SNpc in controls (n = 24) and PD cases (n = 23), normalized to *XPNPEP1* reference gene. (B) Quantification of RIPK1, pRIPK3, pRIPK3/RIPK3, pMLKL, and pMLKL/MLKL protein levels in controls (n = 12) and PD cases (n = 23), normalized to GAPDH and total neuronal density (neurons/mm^2^). (C) Representative Western blots of RIPK1, pRIPK3, RIPK3, pMLKL, and MLKL, and the loading control, GAPDH. The membranes were stripped and reprobed for different protein targets, resulting in duplication of the GAPDH loading control for the same samples. (D) Representative double‐labeling immunofluorescence photomicrographs of the neuronal nuclear protein NeuN (*green fluorescence*) and pRIPK3 (*left, red fluorescence*) or pMLKL (*right, red fluorescence*), with DAPI‐counterstained nuclei (*blue fluorescence*) in snap‐frozen postmortem human SNpc brain sections. All images were taken at 40× magnification. Scale bar = 10 microns.

We next examined markers of necroptosis, including the downstream executor, pMLKL, and kinases (RIPK1, RIPK3, pRIPK3, and MLKL). There was no significant difference in the relative mRNA expression level for *RIPK3* (mean ± SD Control = 3.14 ± 5.68, Int = 0.85 ± 1.16, Adv = 3.52 ± 4.73, *p* = 0.4043; Fig 4A). However, the relative mRNA expression levels for *RIPK1* was reduced by 7.4‐fold in the intermediate‐stage of PD compared with controls (Control = 1.29 ± 0.94, Int = 0.18 ± 0.12, *p* < 0.0001) and increased by 4.0‐fold in advanced‐stage PD compared to intermediate‐stage PD (Adv = 0.70 ± 0.58, *p* = 0.0358; see Fig 4A). Similarly, the relative mRNA expression levels for *MLKL* was reduced by 5.9‐fold in intermediate‐stage PD compared with controls (Control = 3.11 ± 5.29, Int = 0.53 ± 0.91, *p* = 0.0425) and increased by 8.9‐fold in advanced‐stage PD compared to intermediate‐stage PD (Adv = 4.70 ± 6.34, *p* = 0.0113; see Fig 4A).

At the protein level, RIPK1 showed no significant differences between groups (mean ± SD Control = 0.007 ± 0.005, Int = 0.010 ± 0.008, Adv = 0.015 ± 0.010, *p* = 0.0938). Analysis of the active necroptotic kinases, which are involved in the final step of necroptosis, revealed a significant overall difference in the absolute levels of pRIPK3 across the groups (Control = 0.01 ± 0.01, Int = 0.02 ± 0.02, Adv = 0.04 ± 0.03, *p* = 0.0066), with levels being highest in advanced‐stage PD. In contrast, the absolute levels of pMLKL (Control = 0.018 ± 0.015, Int = 0.017 ± 0.017, Adv = 0.026 ± 0.020, *p* = 0.4543) and the activation ratios for both kinases (pRIPK3/RIPK3, Control = 0.08 ± 0.07, Int = 0.24 ± 0.46, Adv = 0.19 ± 0.18, *p* = 0.1867; pMLKL/MLKL, Control = 0.08 ± 0.08, Int = 0.15 ± 0.15, Adv = 0.06 ± 0.04, *p* = 0.1497) were not significantly different (Fig 4B, C). Despite the lack of a significant increase in pMLKL levels in bulk tissue, immunofluorescence demonstrated a small number of pRIPK3+ and pMLKL+ neurons in the PD SNpc at both the intermediate and advanced stages (3 out of 4 PD cases), but none in the 4 controls analyzed (Fig 4D).

### Ferroptotic Signaling is Prominent in Advanced‐Stage PD

To evaluate the activation of ferroptotic signaling, we analyzed key markers representing the 3 core conceptual dimensions of this pathway: iron dyshomeostasis, impaired antioxidant defense, and lipid peroxidation. For iron dyshomeostasis, we measured the protein levels of transferrin receptor 1 (TFR1), which governs cellular iron uptake.^39^ For antioxidant defense, we assessed the expression of GPX4, the central enzyme that neutralizes lipid peroxides and protects against ferroptosis.^40^ To evaluate lipid peroxidation, we measured levels of acyl‐CoA synthetase long‐chain family member 4 (ACSL4), an enzyme that enriches membranes with polyunsaturated fatty acids vulnerable to peroxidation, and 4‐hydroxynonenal (4‐HNE), a key byproduct and marker of lipid oxidative damage. We also assessed nuclear factor erythroid 2‐related factor 2 (NRF2) to investigate the broader cellular antioxidant response.

**FIGURE 5:**
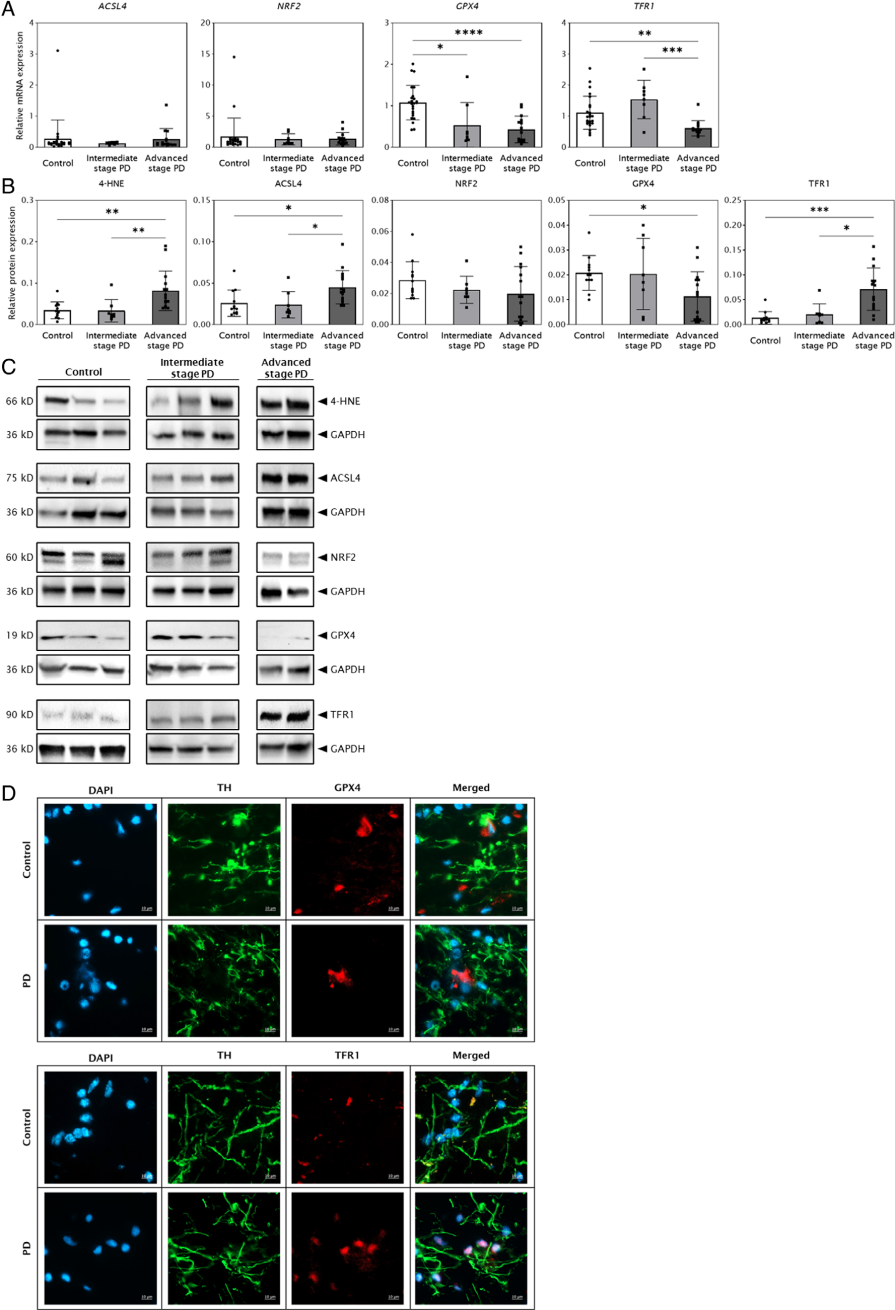
Ferroptotic signaling is potentially more prominent in advanced‐stage Parkinson's disease (PD) substantia nigra pars compacta (SNpc). PD cases are grouped as intermediate‐stage (Braak stages III and IV; n = 8) and advanced‐stage (Braak stages V and VI; n = 15). Data are presented as mean ± SD with Bonferroni adjusted *p* value based on Dunn's Test for multiple comparisons (*p < 0.05; **p < 0.01; ***p < 0.001; ****p < 0.0001). (A) Analysis of relative mRNA levels of the *ACSL4*, *NRF2*, *GPX4*, and *TFR1* genes in the SNpc in controls (n = 24) and PD cases (n = 23), normalized to the *XPNPEP1* reference gene. (B) Quantification of 4‐HNE, ACSL4, NRF2, GPX4, and TFR1 protein levels in controls (n = 12) and PD cases (n = 23), normalized to GAPDH and total neuronal density (neurons/mm^2^). (C) Representative Western blots of 4‐HNE, ACSL4, NRF2, GPX4, TFR1, and GAPDH. The membranes were stripped and reprobed for different protein targets, resulting in duplication of the GAPDH loading control for the same samples. (D) Representative double‐labeling immunofluorescence photomicrographs of tyrosine hydroxylase (TH; *green fluorescence*), and GPX4 (*top panel, red fluorescence*) or TFR1 (*bottom panel, red fluorescence*), with DAPI‐counterstained nuclei (*blue fluorescence*) in snap‐frozen postmortem human SNpc brain sections. All images were taken at 40× magnification. Scale bar = 10 microns.

Compared with controls, *GPX4* mRNA expression significantly decreased by 2.0‐fold in intermediate‐stage PD (mean ± SD Control = 1.08 ± 0.42, Int = 0.53 ± 0.55, *p* = 0.0125) and 2.5‐fold in advanced‐stage PD (Adv = 0.43 ± 0.32, *p* < 0.0001). Similarly, *TFR1* mRNA levels dropped 1.8‐fold in advanced‐stage PD versus controls (Control = 1.11 ± 0.54, Adv = 0.61 ± 0.24, *p* = 0.0028) and 2.5‐fold versus intermediate‐stage PD (Int = 1.54 ± 0.62, *p* = 0.0006), but were unaltered between intermediate‐stage PD and controls (*p* = 0.5780). *ACSL4* and *NRF2* mRNA levels remained unchanged (all *p* values Dunn's adjusted; Fig 5A).

In advanced‐stage PD, GPX4 protein levels were significantly reduced by 45% compared with controls (Control = 0.02 ± 0.01, Adv = 0.01 ± 0.01, *p* = 0.0433). Conversely, TFR1 protein levels were significantly increased 5.2‐fold in advanced‐stage PD versus controls (Control = 0.01 ± 0.01, Adv = 0.07 ± 0.04, *p* = 0.0003) and 3.5‐fold versus intermediate‐stage PD (Int = 0.02 ± 0.02, *p* = 0.0111). Similarly, 4‐HNE levels in advanced‐stage PD were significantly increased 2.4‐fold versus controls (Control = 0.03 ± 0.02, Adv = 0.08 ± 0.05, *p* = 0.0041) and 2.5‐fold versus intermediate‐stage PD (Int = 0.03 ± 0.03, *p* = 0.0042). ACSL4 protein levels in advanced‐stage PD were also significantly increased 1.7‐fold versus controls (Control = 0.03 ± 0.02, Adv = 0.05 ± 0.02, *p* = 0.0176) and 1.9‐fold versus intermediate‐stage PD (Int = 0.02 ± 0.02, *p* = 0.0145). No significant changes were observed for NRF2 protein levels (all *p* values Dunn's adjusted; Fig 5B).

We further assessed whether the expression of ferroptosis markers in advanced‐stage PD correlated with clinical disease duration. Consistent with our findings on neuronal loss, we observed no statistically significant correlation among the protein levels of TFR1, GPX4, NRF2, 4‐HNE, or ACSL4 and disease duration (see Supplementary Fig S1). Moreover, no significant difference was observed between PD cases with longer (≥15 years) versus shorter (<15 years) disease duration (Mann–Whitney *U* test).

Immunofluorescence analysis was performed to visualize the expression of GPX4 and TFR1 within DA neurons of the SNpc. Double‐labeling with TH confirmed changes in these ferroptosis markers within TH+ neurons. Specifically, representative images showed a visible reduction in GPX4 staining intensity and an increase in TFR1 staining intensity within the TH+ neurons of PD cases compared with controls (Fig 5D).

## Discussion

The progressive loss of DA neurons in the SNpc is a cardinal feature of PD, yet the precise mechanisms driving this neurodegeneration remain incompletely understood. This study investigated the contribution of the apoptotic, necroptotic, and ferroptotic cell death signaling pathways in neurons to the characteristic neurodegeneration observed in the PD SNpc by analyzing 47 postmortem brain samples. Our findings confirmed significant neuronal loss in the PD SNpc, which strongly correlated with the advancement of Lewy pathology defined by Braak staging. Our data indicates that canonical caspase‐dependent apoptosis is not a driver of neuron loss in human PD SNpc. Instead, our results highlight the involvement of non‐apoptotic, inflammatory cell death mechanisms. Although evidence for necroptosis activation was present, but limited and variable across cases, we found compelling molecular signatures supporting the activation of ferroptosis, characterized by reduced GPX4 levels and increased markers of iron uptake (TFR1) and lipid peroxidation (4‐HNE and ACSL4). Notably, these ferroptotic changes were more pronounced in advanced‐stage PD compared with intermediate stages, suggesting a stage‐dependent role for this pathway in disease progression.

### Neuronal Loss Correlates With Pathology Progression in PD

The quantitation of neuronal loss in PD has largely been based on studies involving a small number of samples (n < 20), and the underlying mechanisms have not been well‐documented in human postmortem studies. In this study, involving a larger cohort (PD n = 23 and controls n = 12), we showed that 46% of total neurons and 54% of TH+ neurons in the SNpc were lost in PD compared with age‐matched, pathologically healthy controls at the time of postmortem examination. This average reflects stage‐dependent cell death: intermediate‐stage PD (Braak stages III–IV) showed a 29% loss of total neurons and 34% loss of TH+ neurons, whereas advanced‐stage PD (Braak stages V–VI) exhibited a more substantial loss of 56% and 65%, respectively. Similarly, Mazumder et al^41^ also confirmed significant neuron loss in the PD SNpc, albeit reporting a greater extent of loss (70% of total neurons and 69% of DA neurons) based on a smaller sample size (PD n = 6, Controls n = 6). This discrepancy likely reflects the composition of the cohorts; the greater loss observed by Mazumder et al^41^ may be due to their study including only advanced‐stage cases, whereas the inclusion of intermediate Braak stage cases in our study likely lowers the calculated average percentage loss. Furthermore, our findings are also consistent with established literature^8^, ^9^, ^42^ indicating that clinical PD onset can occur when 30 to 40% of total neurons in the SNpc are already lost.

In previous studies, while neuronal loss in the SNpc showed a linear relationship with the severity of PD motor symptoms (Unified Parkinson Disease Rating Scale [UPDRS3]),^8^, ^43^ the correlation of neuronal loss with the PD Braak staging scheme,^32^, ^37^, ^38^ representing the spread of Lewy pathology, had not been documented. In our study, we found that the observed extent of neuronal loss was highly correlated with the PD Braak stages. Therefore, understanding the processes linking α‐synuclein aggregation to neurotoxicity and neuronal dysfunction is expected to be critical for designing disease‐modifying therapies for PD. It is important to note, however, that the relationship between synucleinopathy and PD pathogenesis is complex. Although the Braak staging scheme, based on α‐synuclein deposits, is a critical neuropathological correlate for sporadic PD, it is not without debate and does not represent the full spectrum of the disease.^44^ For instance, a significant proportion of patients with PD, particularly those with young‐onset genetic forms (eg, Parkin and PINK1), exhibit profound dopaminergic degeneration with little to no α‐synuclein pathology.^44^ Furthermore, a disconnect can exist between the burden of Lewy pathology and the extent of neuronal loss, with some cases showing low synucleinopathy despite severe degeneration and vice versa.^44^ Therefore, while our data demonstrate a strong correlation between neuronal loss and Braak staging in this cohort, it underscores the idea that α‐synuclein aggregation is a central marker but not the exclusive driver of neurodegeneration in all forms of PD.

### No Evidence for Apoptosis as a Primary Driver of Neuronal Death

Our comprehensive analysis provides strong evidence against a role for canonical caspase‐dependent apoptosis in PD neurodegeneration. Although protein levels of upstream components like FADD, c‐FLIP, and procaspase‐8 showed an increase in advanced‐stage PD, we observed no corresponding increase in downstream procaspase‐3 levels, and critically, failed to detect the activated forms (cleaved caspase‐8 and cleaved caspase‐3) by either Western blot or immunofluorescence in PD SNpc samples. Furthermore, mRNA levels of key apoptotic effectors (CASP8 and CASP3) were unchanged overall. Although *CFLAR* mRNA decreased and c‐FLIP protein paradoxically increased in advanced‐stage PD (potentially reflecting complex post‐transcriptional regulation or changes in protein turnover under cellular stress), its inhibitory function is consistent with the observed lack of caspase‐8 activation.

These findings corroborate previous reports noting the rarity of morphological and molecular hallmarks of apoptosis in PD brains^14^, ^16^, ^17^, ^18^ and align with studies in other neurodegenerative diseases, including AD and MS, where apoptosis is also not considered the primary neuronal death mechanism,^15^, ^20^, ^21^ but rather TNFR1‐mediated necroptosis is predominant. Overall, apoptosis is primarily a developmental process and/or implicates the withdrawal of trophic support rather than cell intrinsic molecular mechanisms stimulated by neuroinflammation.

### Evidence for Necroptosis in PD

Given the lack of apoptotic signatures, we investigated alternative, regulated cell death pathways. Defined as a pro‐inflammatory form of regulated necrosis executed via the RIPK1‐RIPK3‐MLKL kinase cascade,^19^, ^45^ necroptosis is already implicated in neurodegenerative diseases featuring neuroinflammation, including AD,^15^, ^20^ MS,^21^ and ALS.^22^ Our data on necroptosis markers pointed toward focal activation rather than a widespread event. Although mRNA levels for *RIPK1* and *MLKL* were significantly increased in advanced‐stage relative to intermediate‐stage PD, the protein data provided more direct evidence for pathway activation. We found a significant increase in the absolute level of the active kinase pRIPK3, particularly in advanced‐stage PD. This finding provides biochemical evidence for the activation of the necroptotic cascade. Interestingly, the activation ratio of pRIPK3/RIPK3 was not significantly altered, which may suggest a concurrent increase in the total pool of RIPK3 protein that masks a proportional increase in activation. The discrepancy between this clear upstream activation (pRIPK3) and the lack of a corresponding increase in the downstream executioner (pMLKL) in our bulk analysis is reconciled by our immunofluorescence findings. The presence of small numbers of neurons positive for pRIPK3 and pMLKL in 3 out of 4 PD cases and absent in all 4 controls analyzed suggests that necroptotic signaling, whilst not a uniformly upregulated pathway across all PD stages or cases in our cohort, may be activated in specific neuronal populations or at particular times during the disease course in some individuals and likely driven by the prominent neuroinflammatory environment of the PD SN.

### Evidence for Ferroptosis Activation, Particularly in Advanced‐Stage PD

In contrast to the findings on necroptosis, our data provide compelling evidence for the involvement of ferroptosis in PD, particularly in advanced stages. Ferroptosis is an iron‐dependent, non‐apoptotic cell death pathway marked by lipid peroxidation.^23^, ^24^ This finding is consistent with the progression of Lewy pathology, as α‐synuclein aggregation has been shown to directly induce ferroptosis in dopaminergic neurons. This occurs in part by disrupting iron homeostasis and overwhelming antioxidant systems,^46^ as well as by modulating the membrane's ether‐phospholipid composition to increase vulnerability to lipid peroxidation.^47^ A key piece of converging evidence from our cohort was the significant decrease in both mRNA and protein levels of GPX4, an enzyme that protects against ferroptosis by reducing lipid peroxides.^40^ This downregulation is highly relevant, as GPX4 ablation or inhibition is known to induce ferroptosis and neurodegeneration.^30^, ^48^, ^49^ Concurrently, protein levels of TFR1, responsible for iron uptake, were significantly elevated, particularly in advanced‐stage PD. Furthermore, we detected significantly increased levels of 4‐HNE, a marker of lipid peroxidation, and ACSL4, an enzyme implicated in promoting ferroptosis by enriching membranes with vulnerable fatty acids, again specifically in advanced‐stage PD. The upregulation of ACSL4 is particularly relevant, as its role in promoting ferroptotic neurodegeneration has been demonstrated in both *in vitro* and *in vivo* models of PD.^50^, ^51^ These molecular signatures – decreased GPX4, increased TFR1, ACSL4, and lipid peroxidation – strongly implicate ferroptosis in the neurodegenerative process of PD.

### A Stage‐Dependent Model Integrating Synucleinopathy, Neuromelanin, and Ferroptosis

Our findings, which show focal necroptosis and a predominance of ferroptosis that increases with disease stage, suggest that DA neuron death in PD is not a monolith but a process that evolves. We propose a “2‐hit” model that integrates synucleinopathy and the unique iron‐rich biology of the SNpc.

In the intermediate stage (Braak III–IV), the accumulation of α‐synuclein acts as the “first hit.” Synucleinopathy is a potent trigger for neuroinflammation, activating microglia and driving the pro‐inflammatory environment that would initiate the focal, TNF‐mediated necroptosis we observed (evidenced by pRIPK3). Concurrently, α‐synuclein aggregation itself begins to dysregulate iron homeostasis and sensitize membranes to lipid peroxidation,^46^, ^47^ initiating a baseline level of ferroptosis.

The progression to the advanced stage (Braak V–VI) may be driven by a “second hit” that creates a feed‐forward cycle, making ferroptosis the dominant, runaway pathway. This tipping point is likely the neuromelanin content of SNpc neurons. Neuromelanin is a pigment that avidly sequesters iron; whereas normally protective, this makes SNpc neurons a massive reservoir of iron.^52^ As neurons die from the initial insults (focal necroptosis and early ferroptosis), they release their neuromelanin‐iron complexes into the extracellular space. This neuromelanin is rapidly phagocytosed by microglia. However, microglia cannot degrade neuromelanin, and it has been shown that neuromelanin‐iron accumulation in microglia leads to lysosomal destabilization, rupture, and the catastrophic release of this large iron cargo into the microglial cytosol.^53^ This process not only triggers potent ferroptosis in the microglia themselves but also causes them to release their inflammatory contents and vast, now‐labile iron stores into the microenvironment. This liberated iron is then taken up by adjacent, already‐stressed neurons (via TFR1, which we found is upregulated), overwhelming their already‐depleted GPX4 antioxidant defenses.

This model provides a specific hypothesis for our findings: the moderate, mixed cell death in the intermediate stage transitions to predominant ferroptosis in the advanced stage because this neuromelanin‐driven, microglial‐mediated iron‐release cycle creates a highly toxic, pro‐ferroptotic environment that becomes the primary driver of neurodegeneration.

### Limitations and Future Directions

This study has inherent limitations. First, postmortem tissue analysis provides only a snapshot in time, and correlative findings do not prove causation. Second, acquiring well‐characterized postmortem human brain tissue, particularly from early‐stage PD cases, remains challenging, limiting sample sizes for stratification. Furthermore, detailed clinical data, such as UPDRS or Hoehn and Yahr scores, were not available for this postmortem cohort, precluding a direct correlation between molecular changes and clinical disability. Third, bulk tissue analysis may mask cell‐type‐specific changes, or events occurring in smaller neuronal subpopulations. Finally, although we assessed key markers, the lack of a definitive executioner molecule for ferroptosis highlights the need for more specific biomarkers for this pathway.^54^

Our proposed model has direct implications for future research. It suggests that therapeutic strategies should be stage‐dependent: targeting necroptosis or neuroinflammation may be more effective at earlier stages, whereas ferroptosis inhibitors^55^ or iron chelation therapies may be essential in advanced‐stage disease to break the neuromelanin‐driven iron‐release cycle. Future work using methods like spatial transcriptomics^56^ or single‐cell analyses to probe the neuron–microglia interaction will be needed to validate this hypothesis.

## Conclusions

Through a detailed molecular analysis of apoptosis, necroptosis, and ferroptosis pathways in the postmortem SNpc of PD cases relative to controls, we confirm that significant neuronal loss in the SNpc correlates strongly with the spread of Lewy pathology, as defined by Braak staging. Our findings highlight the likely contribution of non‐apoptotic, inflammatory cell death pathways as the primary drivers of DA neuron death in PD. Although evidence for necroptosis activation was present in a subset of cases, ferroptosis showed stronger and more consistent molecular signatures, including reduced GPX4 and elevated TFR1, ACSL4, and lipid peroxidation markers, particularly in advanced‐stage disease. We propose a model where this progression is driven by an α‐synuclein‐initiated “first hit,” followed by a neuromelanin‐iron‐driven “second hit” that establishes a vicious cycle of ferroptotic cell death. These results provide critical insights into PD pathogenesis and strongly suggest that targeting ferroptosis is a promising therapeutic avenue for slowing neurodegeneration in PD.^55^

## Author Contributions

R.R., J.N.F., and A.J. contributed to the conception and design of the study; A.J. and Y.J.H. contributed to the acquisition and analysis of data; Y.J.H. contributed to drafting the text or preparing the figures.

## Potential Conflicts of Interest

The authors have nothing to report.

## Supporting information


**Supplementary Data S1.** Supporting Information.

## Data Availability

All data generated or analyzed during this study are included in this published article (and its supplementary information files).
