# Supplementary material for: Predominance of Ferroptotic Cell Death Mechanisms in Substantia Nigra Neurodegeneration in Parkinson's Disease
Source: Ann Neurol. 2026 Mar 10;99(6):1415–27. doi: 10.1002/ana.78202 (PMC13206521; doi:10.1002/ana.78202)
Supplement: Supplementary file 1 — Supplementary Data S1. Supporting Information. [file ANA-99-1415-s001.docx]

**Supplementary Table 1: Characteristics of human post-mortem substantia nigra tissue samples**

| **Characteristic** | **Control (n=24)** | **PD Case (n=23)** |  |
| --- | --- | --- | --- |
| **Demographics and Clinical** | | | |
| Age at Death (years, mean / median / range / sd) | 84 / 86 / 69-94 / 7.5 | 79 / 80 / 63-89 / 6.8 |  |
| Age at Onset (years, mean / median / range / sd) | NA | 65 / 67 / 43-82 / 9.0 |  |
| Disease Duration (years, mean / median / range / sd) | NA | 14 / 13 / 5-34 / 7.4 |  |
| Postmortem Interval (hours, mean / median / range / sd) | 33 / 25 / 8-94 / 22 | 19 / 17 / 5-48 / 11 |  |
| Gender | 46% Male (n=11),  54% Female (n=13) | 53% Male (n=13),  47% Female (n=10) |  |
| **Neuropathology (PD Braak Stage)** | | | |
| αSyn Braak Stage 0 | 100% (n=24) | 0% (n=0) |  |
| αSyn Braak Stage III | 0% (n=0) | 13% (n=3) |  |
| αSyn Braak Stage IV | 0% (n=0) | 22% (n=5) |  |
| αSyn Braak Stage V | 0% (n=0) | 17% (n=4) |  |
| αSyn Braak Stage VI | 0% (n=0) | 48% (n=11) |  |

**Supplementary Table 2: Details of primers used for real-time reverse transcriptase quantitative polymerase chain reaction (RT-qPCR)**

| **Gene** | **Assay** | **Unique assay ID** |
| --- | --- | --- |
| *FADD* | PrimePCR™ SYBR® Green Assay: FADD, Human | qHsaCED0046520 |
| *RIPK1* | PrimePCR™ SYBR® Green Assay: RIPK1, Human | qHsaCID0010306 |
| *RIPK3* | PrimePCR™ SYBR® Green Assay: RIPK3, Human | qHsaCED0003731 |
| *MLKL* | PrimePCR™ SYBR® Green Assay: MLKL, Human | qHsaCED0038142 |
| *TFRC* | PrimePCR™ SYBR® Green Assay: TFRC, Human | qHsaCID0022106 |
| *NRF2* | PrimePCR™ SYBR® Green Assay: NFE2L2, Human | qHsaCED0038543 |
| *GPX4* | PrimePCR™ SYBR® Green Assay: GPX4, Human | qHsaCID0023890 |
| *ACSL4* | PrimePCR™ SYBR® Green Assay: ACSL4, Human | qHsaCED0042270 |
| *CFLAR* | PrimePCR™ SYBR® Green Assay: CFLAR, Human | qHsaCID0038905 |
| *CASP8* | PrimePCR™ SYBR® Green Assay: CASP8, Human | qHsaCED0001959 |
| *CASP3* | PrimePCR™ SYBR® Green Assay: CASP3, Human | qHsaCID0013989 |
| *XPNPEP1* | PrimePCR™ SYBR® Green Assay: XPNPEP1, Human | qHsaCID0012153 |

**Supplementary Table 3: Details of primary antibodies used for Western blot and immunostaining**

| **Antigen** | **Host** | **Dilution for Western Blot** | **Dilution for Immunostaining** | **Company** | **Catalogue Number** |
| --- | --- | --- | --- | --- | --- |
| HuC/D | Mouse | NA | 1:500 | Invitrogen Life Technologies,  Ghent, Belgium | A-21271 |
| NEUN | Mouse | NA | 1:1000 | Sigma-Aldrich (Merck),  Darmstadt, Germany | MAB377 |
| TH | Rabbit | NA | 1:200 | Abcam,  Cambridge, UK | AB137869 |
| c-FLIP | Rabbit | 1:1000 | NA | Cell Signalling Technology, Danvers, MA, USA | CST56343S |
| FADD | Rabbit | 1:1000 | NA | Abcam,  Cambridge, UK | AB108601 |
| CASP8 p18 | Rabbit | 1:1000 | NA | Invitrogen Life Technologies,  Ghent, Belgium | PA5-95047 |
| CASP3 p17/19 | Rabbit | 1:1000 | 1:200 | Cell Signalling Technology, Danvers, MA, USA | CST9661S |
| RIPK1 | Rabbit | 1:1000 | NA | Abcam,  Cambridge, UK | AB178420 |
| RIPK3 | Mouse | 1:1000 | NA | R&D Systems,  Minneapolis, MN, USA | MAB7604 |
| MLKL | Mouse | 1:500 | NA | Santa Cruz Biotechnologies, Santa Cruz, CA, USA | SC-293201 |
| pRIPK3 | Rabbit | 1:1000 | 1:500 | Cell Signalling Technology, Danvers, MA, USA | CST93654 |
| pMLKL | Rabbit | 1:1000 | 1:250 | Abcam,  Cambridge, UK | AB187091 |
| 4-HNE | Rabbit | 1:1000 | NA | Abcam,  Cambridge, UK | AB46545 |
| ACSL4 | Mouse | 1:500 | NA | Santa Cruz Biotechnologies, Santa Cruz, CA, USA | SC-271800 |
| NRF2 | Mouse | 1:500 | NA | Santa Cruz Biotechnologies, Santa Cruz, CA, USA | SC-365949 |
| GPX4 | Mouse | 1:1000 | 1:50 | Sigma-Aldrich (Merck),  Darmstadt, Germany | MABF1969 |
| TFR1 | Mouse | 1:1000 | 1:500 | Sigma-Aldrich (Merck),  Darmstadt, Germany | MABC1765 |
| GAPDH | Mouse | 1:1000 | NA | Bio-Rad Laboratories,  Hercules, CA, USA | VMA00046 |

**Supplementary Table 4: Details of secondary antibodies used for Western blot and immunostaining**

| **Secondary Antibody** | **Host** | **Dilution for Western Blot** | **Dilution for Immunostaining** | **Company** | **Catalogue Number** |
| --- | --- | --- | --- | --- | --- |
| Anti-rabbit IgG, HRP-linked Antibody | Goat | 1:10000 | NA | Cell Signalling Technology, Danvers, MA, USA | CST7074S |
| Anti-mouse IgG, HRP-linked Antibody | Goat | 1:10000 | NA | Cell Signalling Technology, Danvers, MA, USA | CST7076S |
| Goat Anti-Rabbit IgG H&L (Alexa Fluor® 488) | Goat | NA | 1:4000 | Abcam,  Cambridge, UK | AB150077 |
| Goat Anti-Mouse IgG H&L (Alexa Fluor® 488) | Goat | NA | 1:4000 | Invitrogen Life Technologies,  Ghent, Belgium | A32723 |
| Goat Anti-Rabbit IgG H&L (Alexa Fluor® 555 | Goat | NA | 1:4000 | Invitrogen Life Technologies,  Ghent, Belgium | A32732 |
| Goat Anti-Mouse IgG H&L (Alexa Fluor® 647) | Goat | NA | 1:4000 | Abcam,  Cambridge, UK | AB150115 |


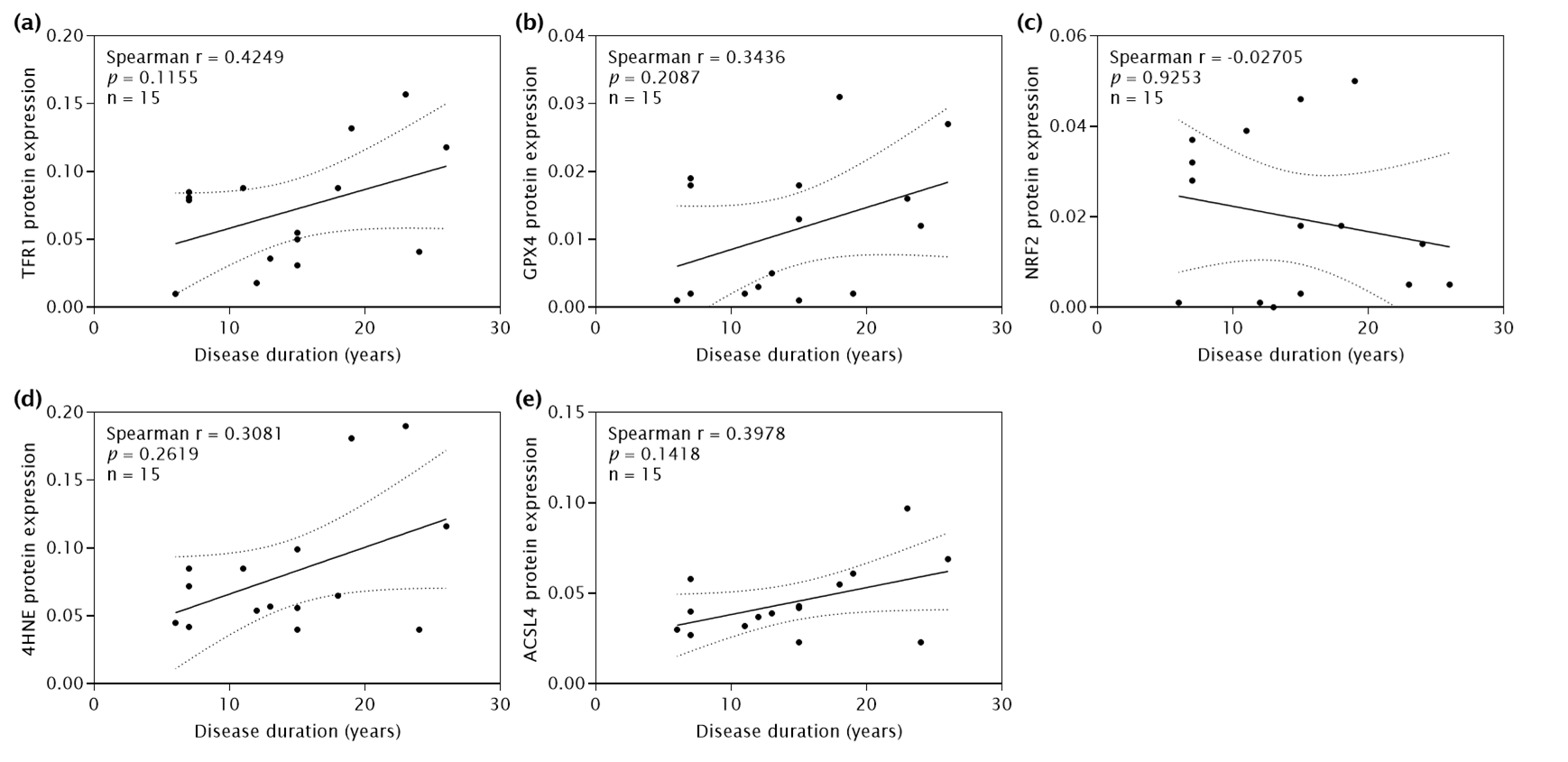


**Supplementary Figure 1. Scatterplot and the corresponding simple linear regression line with 95% confidence interval** showing the relationship between Parkinson’s disease duration (years) and protein expression of ferroptotic markers – (a) TFR1, (b) GPX4, (c) NRF2, (d) 4-HNE, and (e) ACSL4 – in the substantia nigra pars compacta (SNpc) of advanced-stage PD cases (n = 15). Each point represents one advanced-stage PD case. Upper left corner indicates r (Spearman correlation coefficient), *p* (associated *p*-value) and n (sample size).
